# Molecular characterization of low grade and high grade bladder cancer

**DOI:** 10.1371/journal.pone.0210635

**Published:** 2019-01-16

**Authors:** Alessandro Apollo, Valerio Ortenzi, Cristian Scatena, Katia Zavaglia, Paolo Aretini, Francesca Lessi, Sara Franceschi, Sara Tomei, Carlo Alberto Sepich, Paolo Viacava, Chiara Maria Mazzanti, Antonio Giuseppe Naccarato

**Affiliations:** 1 Genetic Unit of Biology Department, University of Pisa, Pisa, Italy; 2 Department of Pathology, University Hospital of Pisa, Pisa, Italy; 3 Section of Cancer Genomics, Fondazione Pisana per la Scienza, Pisa, Italy; 4 Omics Core and Biorepository, Sidra Medicine, Doha, Qatar; 5 Division of Urology, Versilia Hospital, Lido di Camaiore, Italy; 6 Division of Pathology, Hospital of Livorno, Livorno, Italy; Centro Nacional de Investigaciones Oncologicas, SPAIN

## Abstract

**Background:**

Bladder cancer (BC) is the 9^th^ most common cancer diagnosis worldwide. Low grade (LG) represents 70% of all BCs, characterized by recurrence and rare ability (10–15%) to progress to high grade (HG) and invade. The remaining 30% is high grade (HG), fast invasive BC, which is resistant to therapy. Identifying biomarkers for predicting those tumors able to progress is a key goal for patient outcome improvement. This study focuses on the most promising prognostic markers.

**Materials and methods:**

TP53 and FGFR3 mutational status, Survivin, CK19, CK20, E-cadherin and CD44 gene expression analysis were performed on 66 BCs.

**Results:**

Survivin was found associated to tumor grade (p<0.05). Moreover, Survivin correlated with CD44 in TP53 wild type (p = 0.0242) and FGFR3 wild type (p = 0.0036) tumors. In particular the Survivin-CD44 correlation was associated to HG FGFR3 wild type BCs (p = 0.0045). Unsupervised hierarchical clustering based on gene expression data identified four distinct molecular groups reflecting the patient histology (p = 0.038).

**Conclusion:**

We suggest Survivin, both as a biomarker associated to G3 BCs but negatively related to TP53 mutational status, and as a potential novel therapeutic target.

## Introduction

Bladder cancer (BC) is the ninth most common malignant disease and the one of the most common cause of cancer death worldwide [1]. Men are more affected than women (3.5:1 ratio) and BC incidence increases with age differing considerably between geographical regions. European countries (Spain, Italy, Denmark and Switzerland), North America, some northern African countries and western Asia show the highest incidence, while Central and South America, SubSaharan Africa and Southeast Asia show the lowest rates [2]. Cigarette smoke is the main responsible factor for about half of all BCs while several others industrialized chemicals are associated to 20% of BC development [3].

The most common BC symptom is haematuria (microscopic or macroscopic). Macroscopic hematuria is associated with an advanced pathological stage. Unfortunately, microscopic haematuria is not adequately evaluated and there is no active screening for BC [4].

At diagnosis, about 70% of all BCs are low grade (LG) BCs (Ta/pT1/CIS), typically non-invasive tumors, growing as superficial papillary protrusions, genetically associated to FGFR3 mutations and characterized with a high risk of recurrence but low propensity to progress to high grade (HG), invade and metastasize. However, there is a small percentage of LG tumors (10–15%) which are able to progress to HG and become invasive, likely due to acquired TP53 mutations [5, 6]. The remaining 30% of BCs are high grade (HG), genetically associated to TP53 mutations and characterized by a fast direct progression to become invasive tumors (pT2-4). They are mainly flat BCs, developing from severe dysplasia or carcinoma in situ (CIS) and associated to resistance to therapy and poor prognosis. Many genetic factors contribute to tumorigenesis and progression of BC [6, 7], such as mutations in both TP53 and FGFR3 genes [8–9], as well as alterations in the expression of genes involved in cell morphology, epithelial-mesenchimal transformation (EMT) and apoptosis resistance [10], such as CK genes [11], CD44 [12], E-cadherin [13] and Survivin [14].

Currently, tumor, node, and metastasis (TNM) staging and grading systems are insufficient to predict accurately BC evolution. However, the presence of grade 3 (pTa-1G3 or CIS) is the main predictor of progression and mortality in patients affected by non-invasive BC [15]. Predicting which tumors can acquire the susceptibility to progress, invade and/or metastasize is crucial in order to dictate initial therapy and improve the patient outcome. Thus, it is evident a need for tumor markers, to incorporate them into clinical practice adding prognostic information to the conventional TNM and grading systems in terms of treatment response and prognosis [16].

In this study we molecularly characterized 66 histologically non-invasive BCs, considering the most promising prognostic markers. We analyzed TP53, and FGFR3 gene mutational status and gene expression of Survivin, CK19, CK20, CD44 and E-cadherin. Especially, we performed association analysis of the target genes within high grade (HG) and low grade (LG) BCs. Then, we focused on comparative analysis of the molecular status with tumor grade and morphology (papillary or flat) in order to characterize those BCs with high propensity to progress such as HG flat G3 (CIS) underlining putative molecular targets to therapy. Furthermore, we investigated a possible correlation among genes and performed hierarchical clustering analysis in order to find in our population a stratification driven solely by the molecular status.

## Materials and methods

### Samples collection

A total of 66 BC samples: 58/66 were male and 8/66 female with an mean age of 75. The patient population was composed of 48 formalin-fixed paraffin embedded (FFPE) tissues and 18 fresh tissues, collected at the Versilia Hospital (Viareggio, Italy). All 66 samples were histologically non-invasive. 33 out of 66 were histologically diagnosed as HG, and 33 as LG tumours (Table 1 and Table A in S1 Tables).

**Table 1:** Histological feature of the study set.

histogy	n° patient	FFPE	Fresh Tissue
LG	33	21	12
HG	33	27	6
**TOT**	66	48	18

Clinical feature of the study set. LG: Low Grade. HG: High Grade. FFPE: Formalin-fixed paraffin embedded tissues.

**Fig 1:**
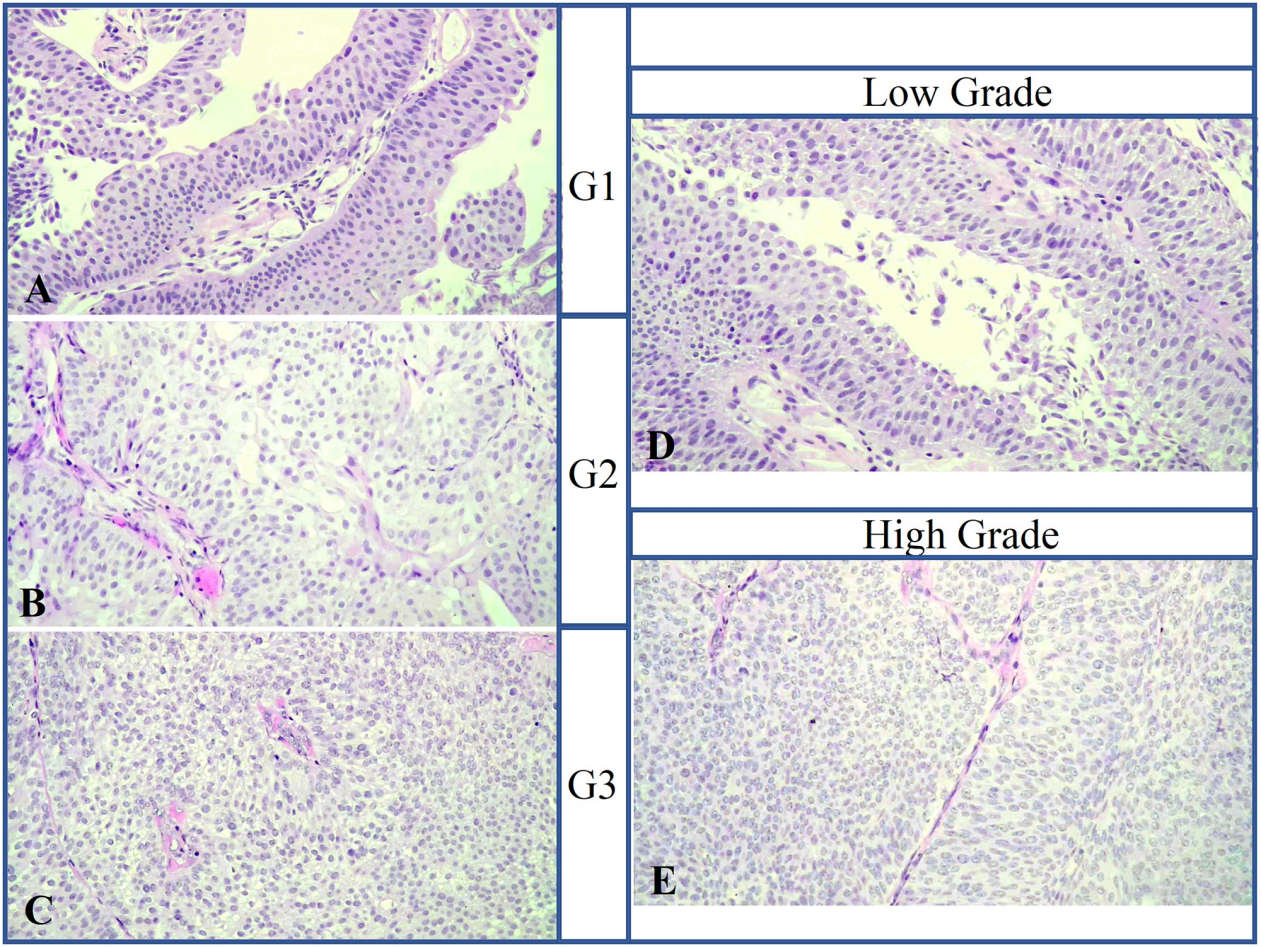
Example of the histologic classification of G1 (A), G2 (B) and G3 (C) as well as LG (D) and HG (E) BCs among study cases (original magnification x20).

A detailed grading was available only for the 48 FFPE samples: 9/48 were G1 (pTa), 13/48 were G2 (pTa) and 26/48 were G3. In the G3 group 7/26 showed papillary morphology (pTa) while the remaining 19 had no papillary morphology (pTis) at histological diagnosis (Table 2 and Table A in S1 Tables). An example of the histologic classification of G1 (A), G2 (B) and G3 (C) as well as LG and HG BCs among our cases is showed in Fig 1. The study was approved by the Ethics Committee of the University Hospital of Pisa and all methods were performed in accordance with approved guidelines.

**Table 2:** Histological grading and morphology of BC patients.

Grade	Histology	Stage	Morphology	n° patients
G1	LG	pTa	Papillary	9
G2	LG	pTa	Papillary	13
G3	HG	pTa-T1	Papillary	7
G3	HG	pTis	Non Papillary/Flat	19
**TOT**				48

Histological grading and morphology of BC patients. LG: Low Grade. HG: High Grade. G1: Grade 1; G2: Grade: 2; G3: Grade 3.

### TP53 and FGFR3 gene mutational analysis

DNA extracted from 4 x 10 μm FFPE tissue sections (Macherey-Nagel, Düren, Germany) was used to amplify TP53 exon 4–9 PCR and FGFR3 exon 7, 10, 15 by PCR (Applied Biosystem, MA). PCR product was purified (Millipore) and analyzed by ABI-XL 3130 Sequencer (Applied Biosystem, MA USA). Because of the poor quality of the DNA extracted from the FFPE tissues we could not perform the TP53 and FGFR3 mutational status on all the samples.

### Gene expression analysis of Survivin, CK20, CD44 and E-cadherin

Total RNA extracted from 4 x 10 μm FFPE tissue sections (Helix Extraction System, Diatech) was reverse transcribed in cDNA (Invitrogen, Carlsbad, CA). Gene expression levels were analyzed by Rotor-Gene 6000 (Corbett, Qiagen, CA). Endogenous reference gene (beta 2 microglobulin) was used to normalize each gene expression level (Table C in S1 Tables). Because of the poor quality of the RNA extracted from the FFPE tissues we could not perform the gene expression analysis on all the samples.

### Statistical analysis

*Chi-square and Fisher tests* were used to analyze the frequencies of TP53 and FGFR3 genetic alteration, *while t-student* was performed to study the differential gene expression in the patient population studied.

*Unsupervised Hierarchical clustering analysis* considering all the gene expression data was performed by Tanagra Software.

*Multivariable Analysis* was used to investigate a correlation among genes taking into account the effects of all histological and morphological variables. p-value ≤ 0.05 indicates statistical significance of all the analysis (StatGraphics XVI software).

## Results

### TP53-FGFR3 gene mutational status in BC samples

TP53 and FGFR3 somatic mutations were detected in 35.6% (21/59) and 31% (18/58) of the samples, respectively. Both TP53 and FGFR3 mutational profiles showed a heterogeneous distribution between LG and HG BCs. TP53 mutations were in 40.7% (11/27) of LG while in 31.2% (10/32) of HG tumors. FGFR3 mutations were in 37% (10/27) and 25.8 (8/31) of LG and HG tumors respectively. FGFR3 and TP53 mutations were mutually exclusive: co-occurrence of TP53-FGFR3 mutations was only in 5.2% (3/58) of the BC samples: 2/27 of LG and 1/31 in HG tumors. (Table 3).

**Table 3:** Distribution of TP53 and FGFR3 somatic mutations in LG and HG tumors.

Histology	TP53	FGFR3	TP53-FGFR3 co-occurence
**LG**	40.7% (11/27)	37% (10/27)	7.4% (2/27)
**HG**	31.2% (10/32)	25.8% (8/31)	3.2% (1/31)
**ALL BCs**	35.6% (21/59)	31% (18/58)	5.2% (3/58)

Distribution of TP53 and FGFR3 somatic mutations in tumors. LG: Low Grade; HG: High Grade. BC: Bladder Cancer.

Interestingly within HG tumors, TP53 mutations were strongly associated to flat morphology compared to papillary one, being detected in 33.3% (9/27) and 16.5% (1/6) of flat and papillary tumors respectively (data not statistically significant). On the other hand, FGFR3 mutation distribution did not strongly change between the two groups. FGFR3 mutations were in 22.2% (6/27) and 33.3% (2/6) of flat and papillary HG tumors, respectively (Table 4). Among all the HG BCs, only one flat tumor showed TP53-FGFR3 overlapped mutations.

**Table 4:** Distribution of TP53 and FGFR3 somatic mutations in relation to tumor morphology and histology.

Histological Grading and morphology	TP53	FGFR3	TP53-FGFR3 co-occurence
**papillary LG**	45.5% (11/22)	27.2% (6/22)	9% (2/22)
**papillary HG**	16.7% (1/6)	33.3% (2/6)	0% (0/6)
**flat HG**	33.3% (9/27)	22.2% (6/27)	3.7% (1/27)

Distribution of TP53 and FGFR3 somatic mutations in relation to tumor morphology and histology. LG: Low Grade; HG: High Grade.

Among the TP53 mutations, Q52STOP was the most frequent with a percentage of 3.6% (1/28) and 7.6% (2/26) in LG and HG respectively (Table 5). FGFR3 common mutations such as R248C, S249C and Y375C were in 5% (3/60), 3.4% (2/60) and 5% (3/60) of the BCs respectively (Table 6). We identified six unknown mutations for TP53, while seven for FGFR3 gene. The detailed distribution of the genetic alterations detected in both TP53 and FGFR3 is showed in Tables 5 and 6, as well as Table B in S1 Tables.

**Table 5:** Detailed distribution of TP53 somatic mutations in LG and HG BCs.

TP53	LG	%	HG	%
Known Mutations	P36L: CCG-CTG	3.6% (1/28)	Q52STOP: CAA-TAA	7.6% (2/26)
	S46F: TCC-TTC	3.6% (1/28)	P152R: CCG-CGG	3.8%(1/26)
	Q52STOP: CAA-TAA	3.6% (1/28)	Q167STOP: CAG-TAG	3.8%(1/26)
	W53STOP: TGG-TGA	3.6% (1/28)	H179R: CAT-CGT	3.8%(1/26)
	S116C: TCT-TGT	3.2% (1/31)	Y205C: TAT-TGT	3.4% (1/29)
	T150I: ACA-ATA	3.8% (1/26)	S215C: AGT-TGT	3.4% (1/29)
	Q167: STOP: CAG-TAG	3.8% (1/26)	R273S: CGT-AGT	3.4% (1/29)
	H168Y: CAC-TAC	3.8% (1/26)	A276V: GCC-GTC	3.3% (1/30)
	P177L: CCC-CTC	3.8% (1/26)	R290C: CGC-TGC	3.4% (1/29)
	D184N: GAT-AAT	3.8% (1/26)		
	A276V: GCC-GTC	3.4% (1/29)		
	R280T: AGA-ACA	3.1% (1/31)		
	R306STOP: CGA-TGA	3.4% (1/29)		
Unknown Mutations	D57V: GAC-GTC	3.6% (1/28)		
	P64L: CCC-CTC	3.6% (1/28)		
	P36S: CCG-TCG	3.6% (1/28)		
	R65K: AGA-AAA	3.6% (1/28)		
	A178T: GCC-ACC	3.8% (1/26)		
	T231I: ACC-ATC	3.4% (1/29)		
Insersions			C.622-623_insG(STOP)	3.8% (1/26)

Detailed distribution of TP53 somatic mutations in LG and HG BCs. HG and LG tumors. LG: Low Grade; HG: High Grade.

**Table 6:** Detailed distribution of FGFR3 somatic mutations in LG and HG BCs.

FGFR3	LG	%	HG	%
Known Mutations	R248C: CGC-TGC	10% (3/30)	S249C: TCC-TGC	6.7% (2/30)
	G372C: GGC-TGC	3% (1/30)	A265T:GCG-ACG	3% (1/30)
	Y375C: TAT-TGT	6.7% (2/30)	Y375C: ATA-TGT	3% (1/30)
	F386L: TTC-CTC	3% (1/30)	P404L: CCC-CTC	3% (1/30)
	L378F: CTC-TTC	6.7% (2/30)	G405D: GGC-GAC	3% (1/30)
	P403S: CCC-TCC	3% (1/30)		
	P304L: CCC-CTC	6.7% (2/30)		
Unknown Mutations	L259P: CTG-CCG	3% (1/30)	S249F: TCC-TTC	3% (1/30)
	H284Y: CAC-TAC	3% (1/30)	Q263R: CAG-CGG	3% (1/30)
			H290Y: CAC-TAC	3% (1/30)
			A368T: GCT-ACT	3% (1/30)
			S373N: AGT-AAT	3% (1/30)

Detailed distribution of FGFR3 somatic mutations in LG and HG BCs. LG: Low Grade. HG: High Grade.

### Association analysis of gene espression levels with LG and HG BCs

**Fig 2:**
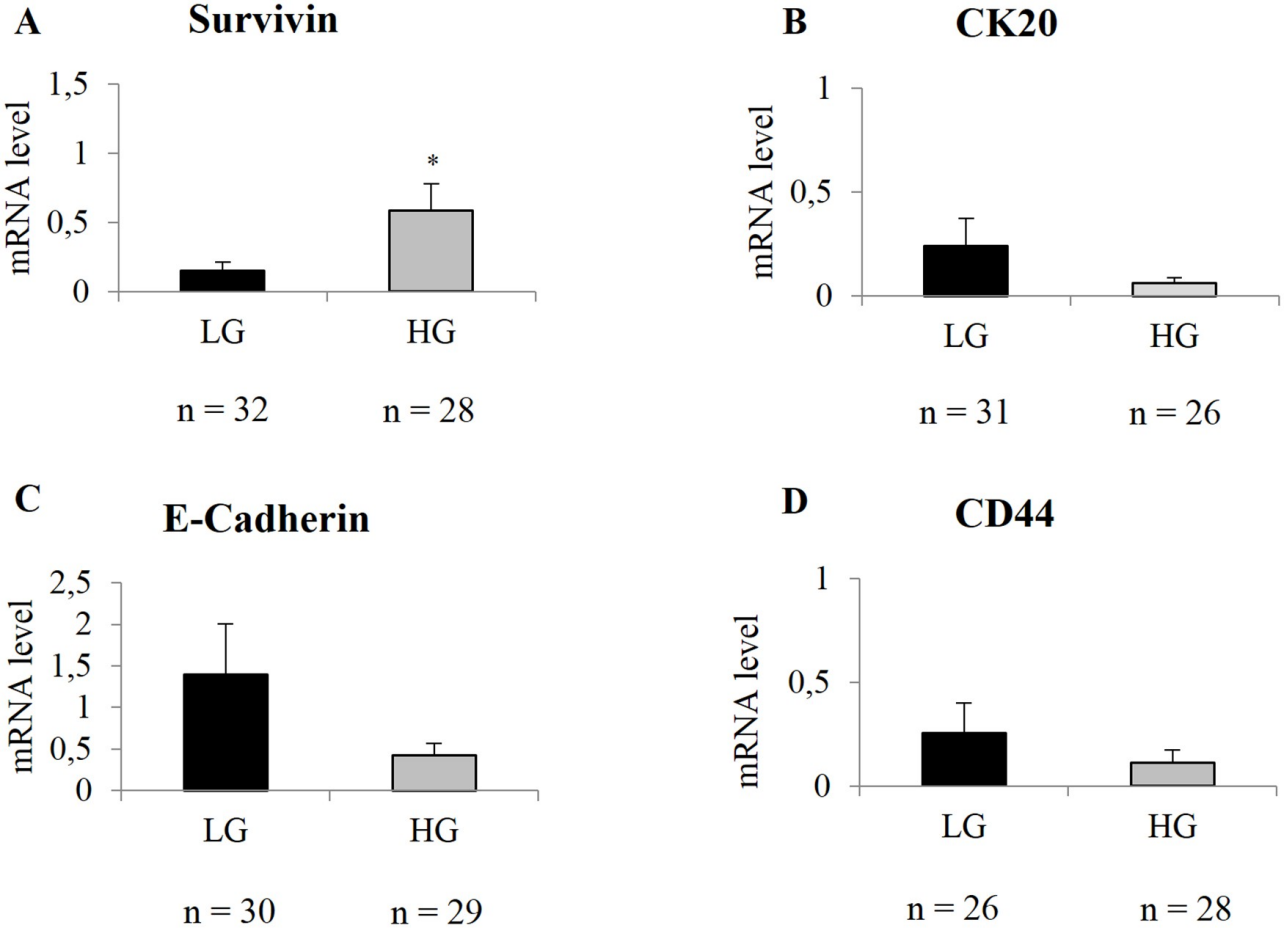
Differential gene expression analysis between HG and LG tumors. Survivin (A), CK20 (B), E-Cadherin (C) and CD44 (D) gene expression levels in LG and HG BCs. LG: Low Grade; HG: High Grade. Standard error of the mean (SEM) is indicated by the bars. n indicates the number of analyzed samples. * indicates p-value <0.05.

To study the relationship between the molecular profile and histological classification (in terms of low-high grade), we analyzed, in HG and LG BCs, the expression of genes involved in BC development, such as Survivin, CK19, CK20, CD44 and E-cadherin, (Fig 2). Survivin gene expression was significantly overexpressed (p = 0.04) in HG, showing mRNA levels 3.8 fold higher than LG (mRNA levels: 0.58 ± 0.19 and 0.15 ± 0.06 respectively) (Fig 2A). HG tumors showed a four fold down-regulation of CK20 compared to LG BCs (mRNA levels: 0.24 ± 0.13 and 0.06 ± 0.02 respectively) (Fig 2B). CK19 mRNA levels slightly decreased (data not showed) between LG and HG tumors (mRNA levels: 27.09 ± 5.53 and 18.22 ± 4.12 respectively). E-cadherin mRNA level was 3.3 fold higher in LG than HG group (mRNA level: 1.34 ± 0.61 and 0.44 ± 0.14 respectively), while CD44 showed an over-expression of 2.1 fold in LG compared to HG tumors (mRNA levels: 0.24 ± 0.14 and 0.11 ± 0.06 respectively) (Fig 2C and 2D). However CK19 (data not showed), CK20, E-cadherin and CD44 gene expression analysis were not significant.

**Fig 3:**
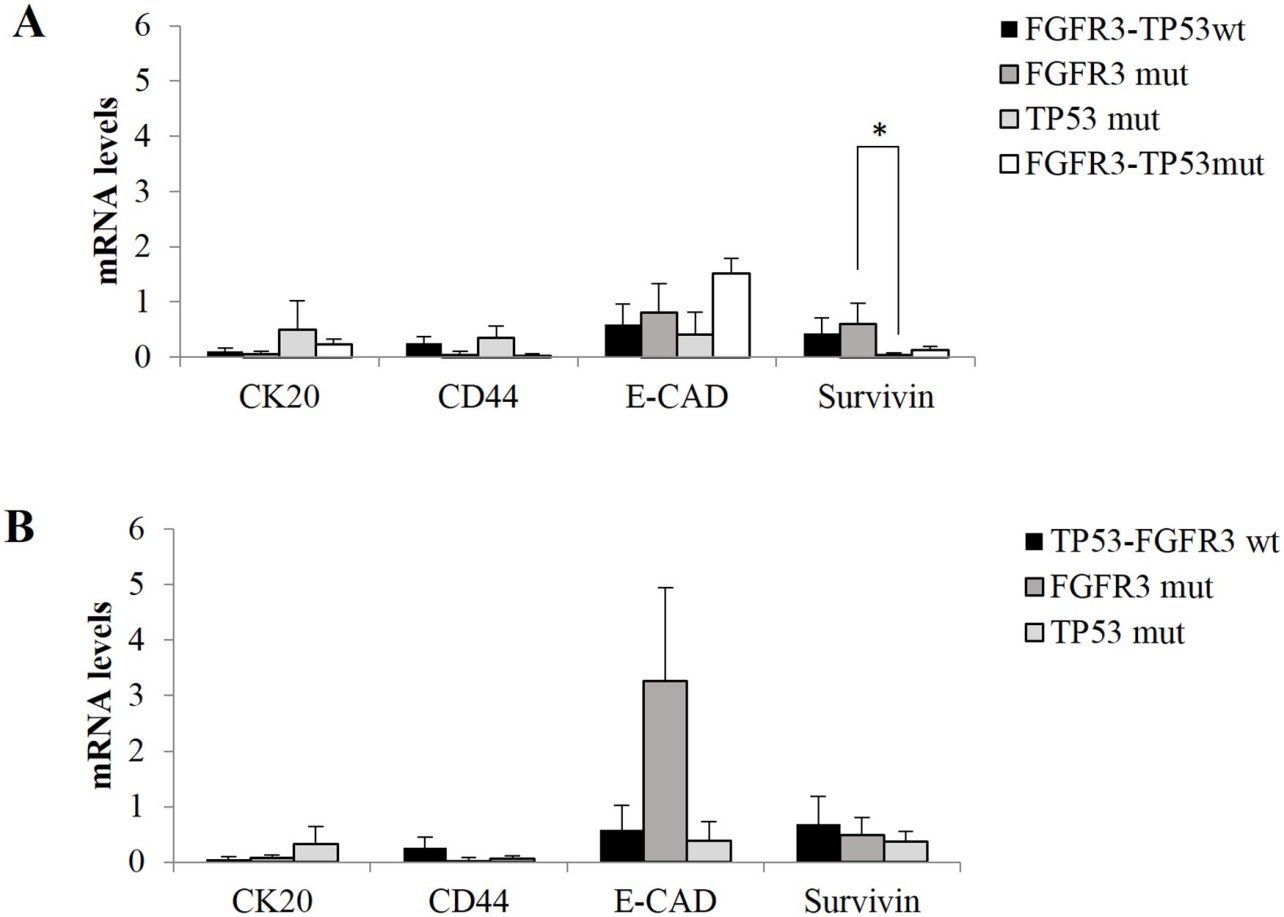
Differential gene expression of CK20, CD44, E-cadherin, and Survivin in relation to FGFR3 and TP53 mutational status of LG and HG BCs. CK20, E-cadherin, CD44 and Survivin mRNA levels in TP53-FGFR3 wild type, FGFR3 mutated, TP53 mutated and FGFR3-TP53 mutated LG tumors (A). CK20, E-cadherin, CD44 and Survivin in TP53-FGFR3 wild type, FGFR3 mutated and TP53 mutated HG tumors (B). Only one HG tumor showed overlapped TP53-FGFR3 mutations. It is no sufficient for any statistical analysis. CK20: cytokeratin 20; E-CAD: E-cadherin: Low Grade; HG: High Grade. Standard error of the mean (SEM) is indicated by the bars. n indicates the number of analyzed samples. * indicates p-value <0.05.

Then, withinin LG and HG tumors, we investigate the relationship between the expression of these target genes and the TP53-FGFR3 mutational status of the tumor. We observed that Survivin expression seems to be inversely related to TP53 mutational status, in LG tumors. TP53 mutations, alone or in combination with FGFR3 mutations, were associated to a decrease in Survivin mRNA levels compared to wild type LG BCs. In particular TP53 mutated tumors had 12.7 times and 17.5 times decrease of Survivin mRNA levels compared to wild type (data not significant) and FGFR3 mutated LG BCs (p = 0.021), respectively (Fig 3A). On the other hand, FGFR3 mutated and TP53-FGFR3 double mutated BCs showed 9 fold decrease of CD44 mRNA levels compared to both, wild type and TP53 mutated tumors (dat not significant), respectively. In details, Survivin mRNA levels were 0.43 ± 0.28, 0.59 ± 0.38, 0.034 ± 0.04 and 0.12 ± 0.06 in wild type, FGF3 mutated, TP53 mutated and FGFR3-TP53 mutated LG tumors respectively. CD44 was 0.25 ± 0.11, 0.04 ± 0.06, 0.34 ± 0.21 and 0.03 ± 0.03, CK20 was 0.10 ± 0.06, 0.57 ± 0.042, 0.49 ± 0.53 and 0.20 ± 0.10, E-Cadherin was 0.59 ± 0.36, 0.8 ± 0.52, 0.41 ± 0.4 and 1.51 ± 0.28 in wild type, FGF3 mutated, TP53 mutated and FGFR3-TP53 mutated LG tumors respectively (Fig 3A). Down-regulation of Survivin correlated with TP53 mutational status was confirmed also in HG, compared to both, wild type and FGFR3 mutated tumors. In addiction, TP53 tumors showed 4 fold increase of CK20 mRNA compared to wild type tumors, not significanlty (Fig 3B).

**Fig 4:**
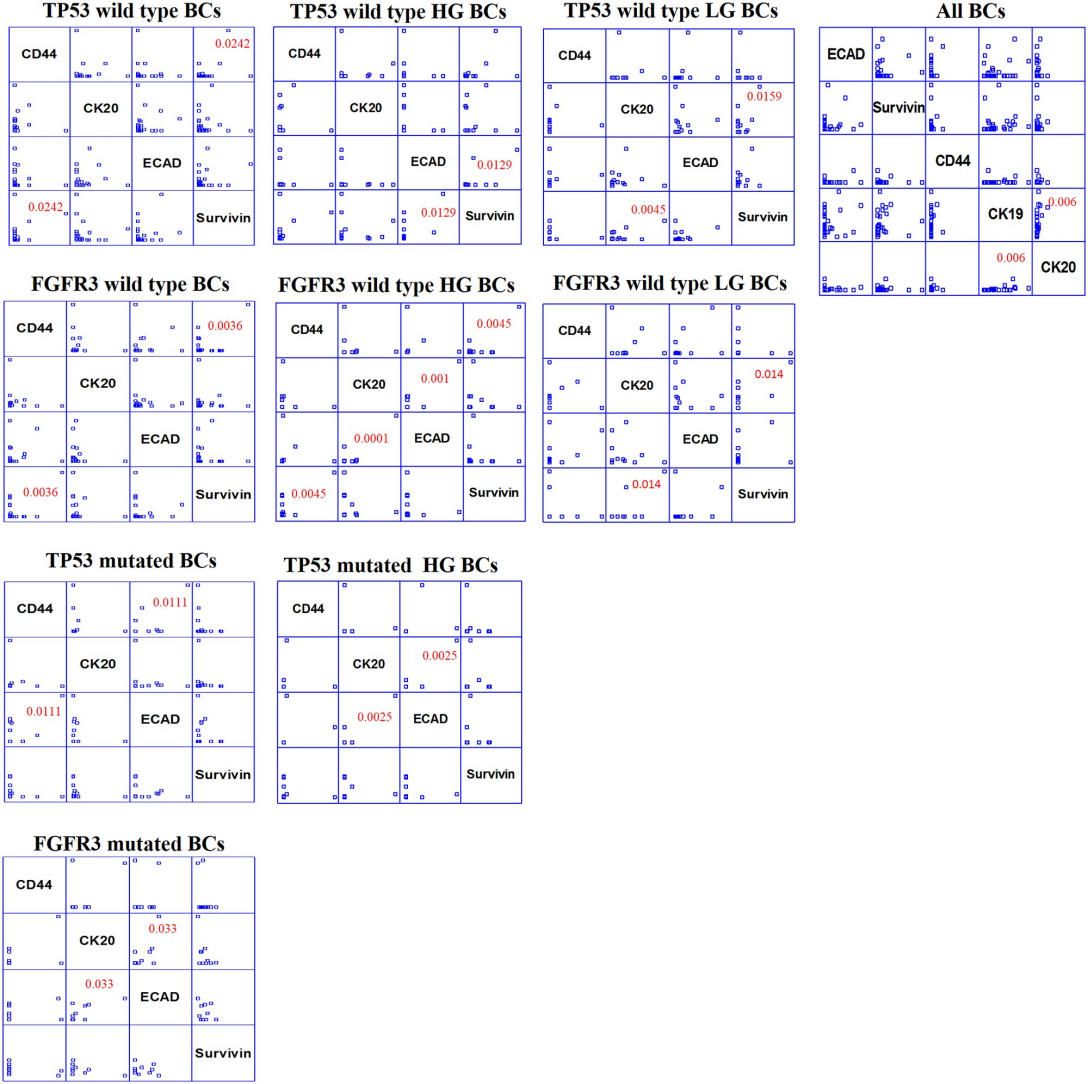
Intergene multivariable analysis. Multivariable analysis of CD44, CK20, E-cadherin (ECAD) and Survivin in all BCs, HG and LG BCs taking in account also the mutational status of both TP53 and FGFR3 genes. To underline the statistically significant correlations, p-value is reported in red. (StatGraphics XVI software).

Moreover multivariable analysis showed Survivin and CD44 strongly correlated each other in both TP53 wild type (p = 0.0242) and FGFR3 wild type (p = 0.0036) BCs, whereas E-cadherin correlated with CD44 exclusively in TP53 mutated (p = 0.011) tumors, as well as with CK20 only in FGFR3 mutated (p = 0.033) BCs. Within HG tumors, E-cadherin correlated with CK20 in those BCs harboring TP53 mutations (p = 0.0025), as well as with Survivin in TP53 wild type HG tumors (p = 0.0129). FGFR3 wild type HG tumors showed Survivin and CD44 significantly correlated each other (p = 0.0045) as well as CK20 and E-cadherin (p = 0.0001). On the other hand, within LG BC, Survivin and CK20 correlated each other in TP53 wild type (p = 0.0159) or FGFR3 wild type (p = 0.0104) tumors. Multivariable analysis on the total population of patients showed a positive significant correlation between CK19 and CK20 (p = 0.006) (Fig 4).

### Association analysis of gene expression levels with BC molecular grading

**Fig 5:**
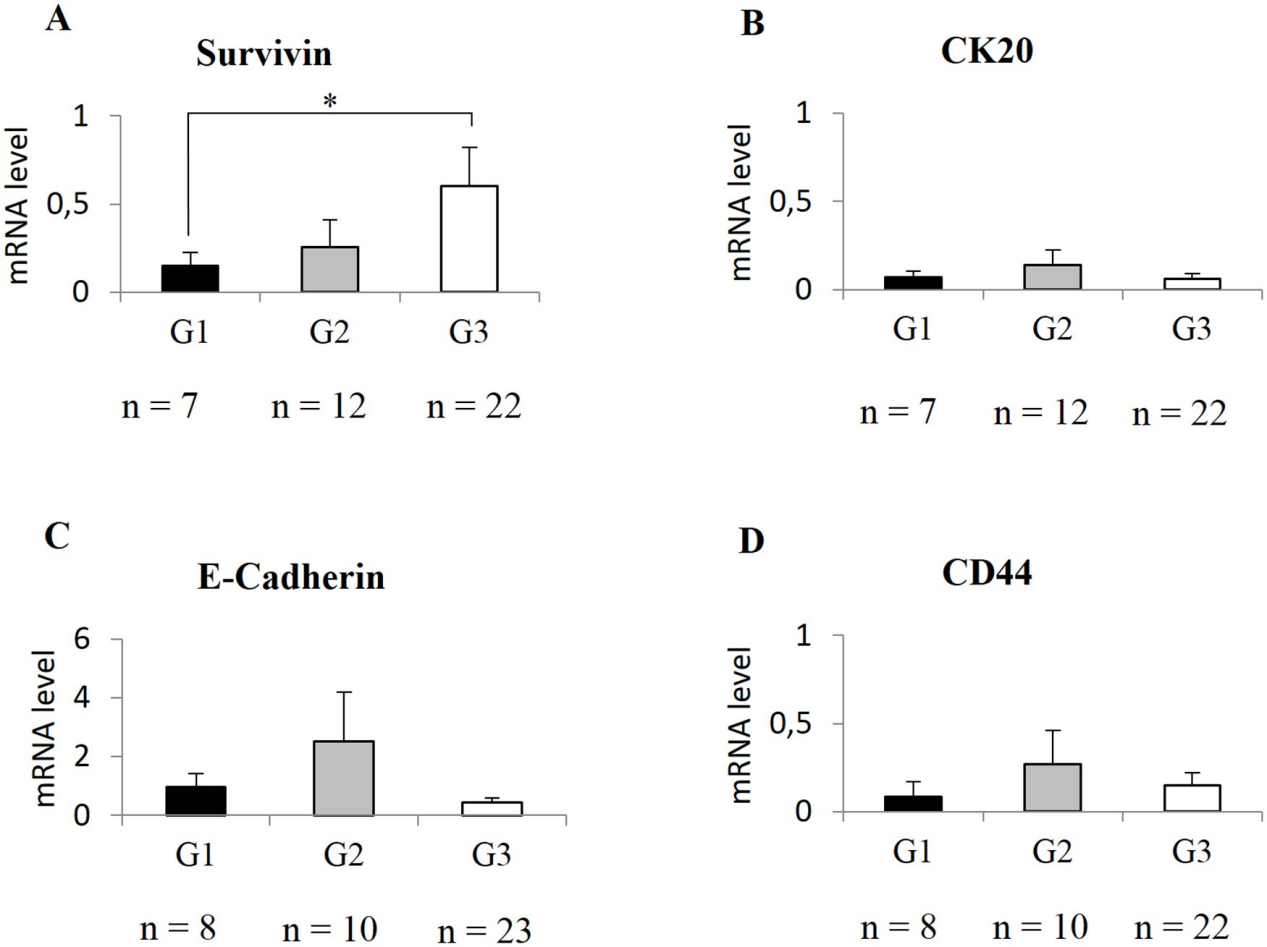
Differential gene expression analysis within tumor grading. Survivin (A), CK20 (B), E-cadherin (C) and CD44 (D) gene expression levels in G1, G2 and G3 BCs. G1: Grade 1; G2: Grade 2; G3: Grade 3. Standard error of the mean (SEM) is indicated by the bars. n indicates the number of analyzed samples. * indicates p value < 0.05.

To deeply investigate the relationship between markers gene expression levels and tumor grade, we analyzed the mRNA expression levels of Survivin, CK19, CK20, CD44 and E-Cadherin genes in BCs G1, G2 and G3. Among all these markers only Survivin was significantly overexpressed in G3 compared to G1 tumors (p = 0.04), showing mRNA levels of 0.15 ± 0.07, 0.25 ± 0.15, 0.60 ± 0.21 in G1, G2 and G3 respectively (Fig 5A). CK20, E-cadherin and CD44 gene expression resulted to be the highest in the G2 group, while G1 and G3 had comparable mRNA levels. CK20 mRNA levels was 0.07 ± 0.03, 0.13 ± 0.08, 0.06 ± 0.03 in G1, G2 and G3 respectively (data not statistically significant) (Fig 5B). E-cadherin mRNA level was 0.98 ± 0.44, 2.5 ± 1.69, 0.45 ± 0.14 in G1, G2 and G3 respectively while CD44 mRNA level was of 0.08 ± 0.08, 0.27 ± 0.19, 0.15 ± 0.07 in G1 G2 and G3 (data not statistically significant) (Fig 5C and 5D). CK19 gene expression has not changed significantly among groups (data not showed).

### Association analysis of gene expression levels with BC morphology

**Fig 6:**
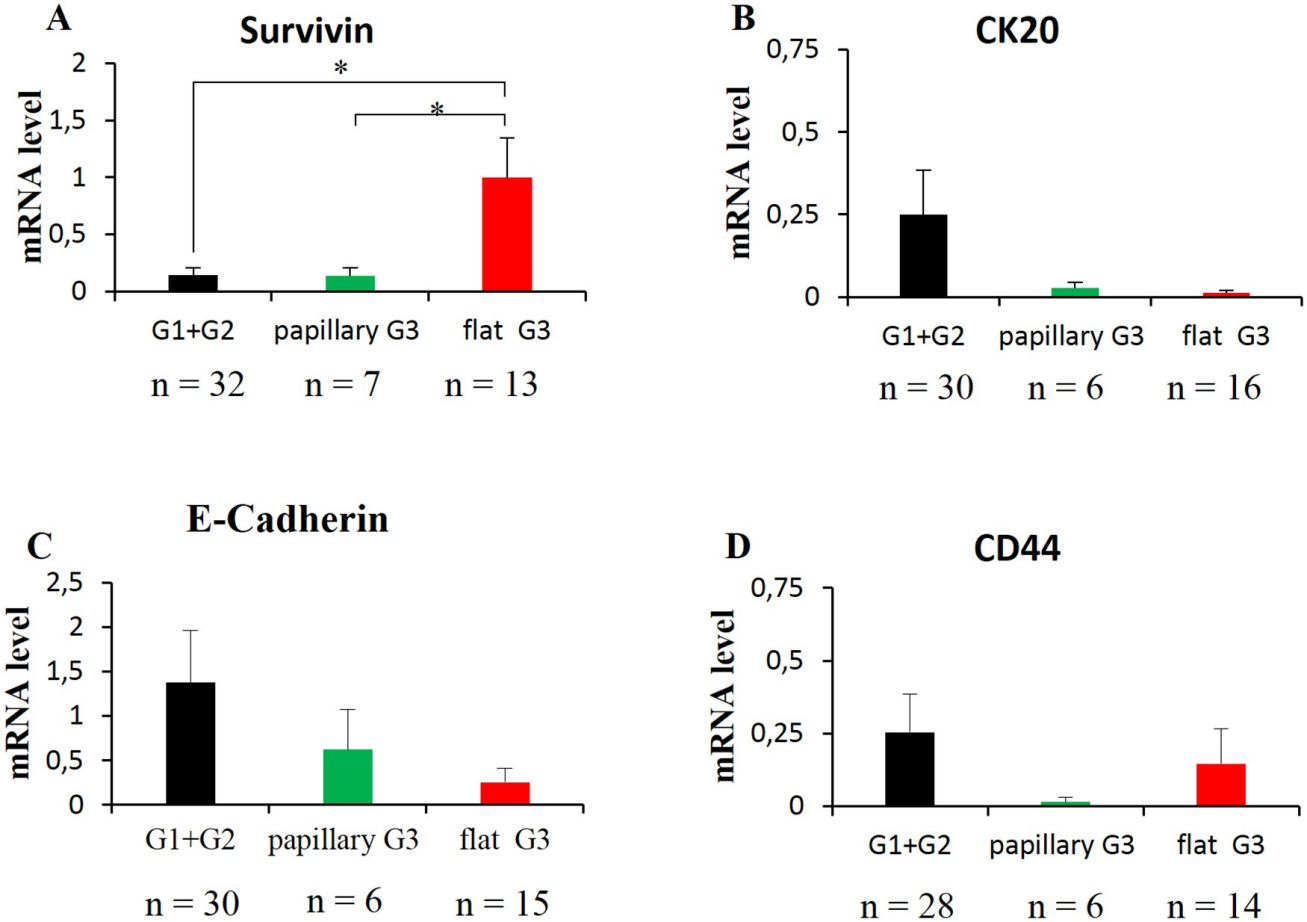
Differential gene expression analysis within BC morphology. Survivin (A), CK20 (B), E-cadherin (C) and CD44 (D) gene expression levels in the BCs subtypes defined by the BC morphology. G1+G2 were LG with papillary morphology; papillary G3 were HG characterized as papillary protrusion; flat G3 were HG with non papillary morphology. Standard error of the mean (SEM) has indicated by the bars. n indicates the number of analyzed samples. * indicates p value < 0.05.

To evaluate the relationship between gene expression markers, tumor grade and morphology, we further stratified our BC population on the basis of tumor grade and morphology at diagnosis. We defined LG tumors presenting papillary morphology (G1+G2), HG with papillary morphology (papillary G3) and non-papillary HG (flat G3). mRNA levels of Survivin, CK20, E-cadherin and CD44 are shown in Fig 6. Survivin showed a statistically significant up-regulation (from 7.14 to 7.7 fold) in flat G3 compared with both G1+G2 and papillary G3 groups (p = 0.017 and 0.016 respectively). G1+G2 and papillary G3 groups showed no difference in Survivin mRNA levels. Survivin mRNA level was 0.14 ± 0.06, 0.13 ± 0.06, and 1.00 ± 0.34 in G1+G2, papillary G3 and flat G3 respectively (Fig 6A). CK20 mRNA levels were strongly reduced (from 8.3 to 25 fold) in both papillary G3 and flat G3 groups compared to G1+G2 group (not statistically significant). CK20 mRNA levels were 0.25 ± 0.13, 0.03 ± 0.01, and 0.01 ± 0.008 in G1+G2, papillary G3 and flat G3 respectively (Fig 6B). E-cadherin mRNA levels were 1.37 ± 0.59, 0.62 ± 0.42, and 0.25 ± 0.16, while CD44 showed mRNA levels of 0.25 ± 0.13, 0.01 ± 0.01 and 0.15 ± 0.12 in G1+G2, papillary G3 and flat G3 respectively (not statistically significant) (Fig 6C and 6D). CK19 gene expression has not change significantly among the groups (data not showed).

### Hierarchical clustering analysis

**Fig 7:**
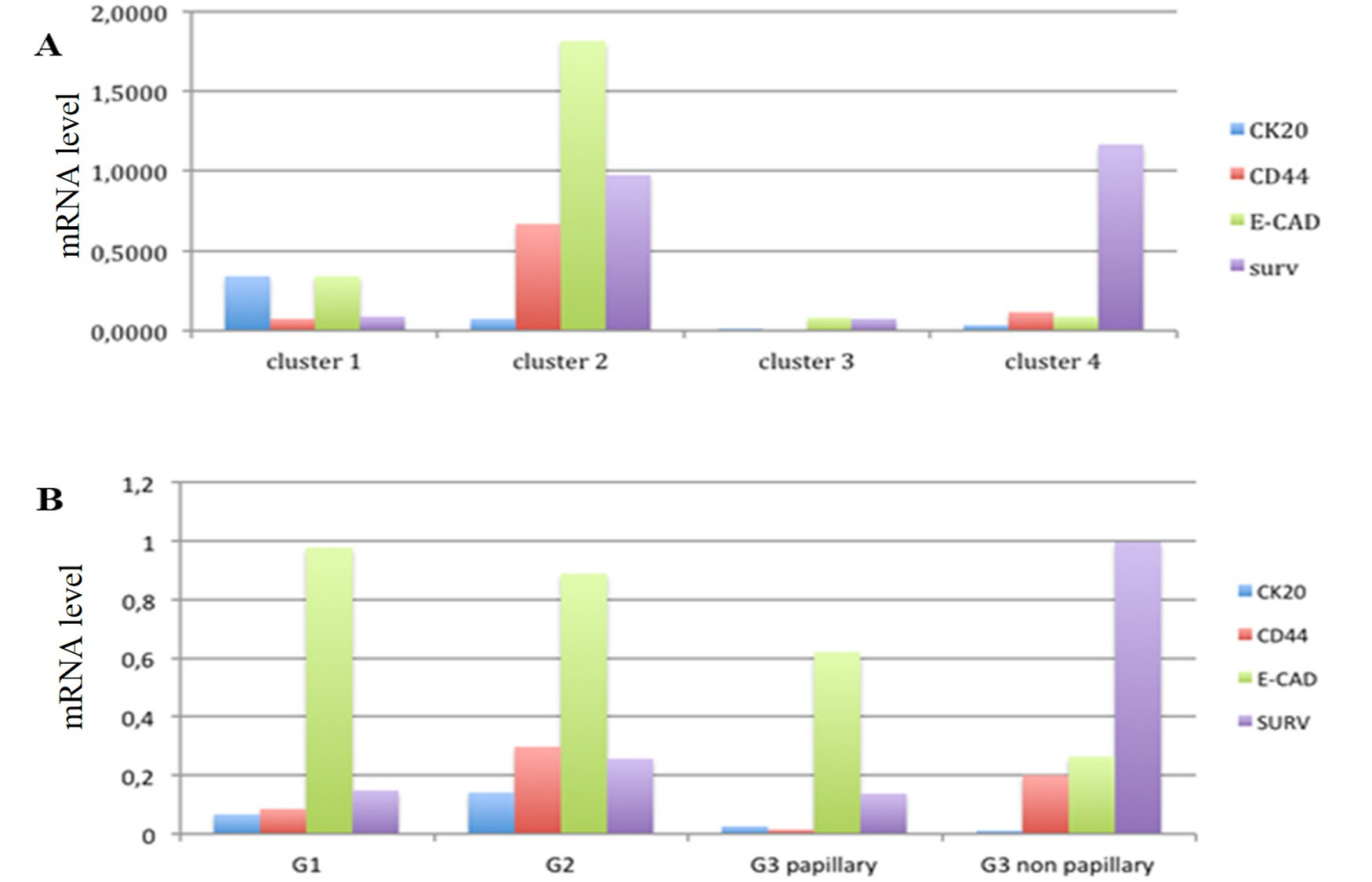
Clustering analysis. Unsupervised clustering analysis combining gene expression levels of CK20, CD44, E-CAD and Survivin genes created 4 distinct clusters (A). Gene expression levels of CK20, CD44, E-CAD and Survivin genes in the clustering of BCs defined by their grading and morphology (B). It is possible to identify similar trends in gene expression profile between Cluster 2 and G2 group and Cluster 4 and G3 non papillary (G3 flat) group.

**Fig 8:**
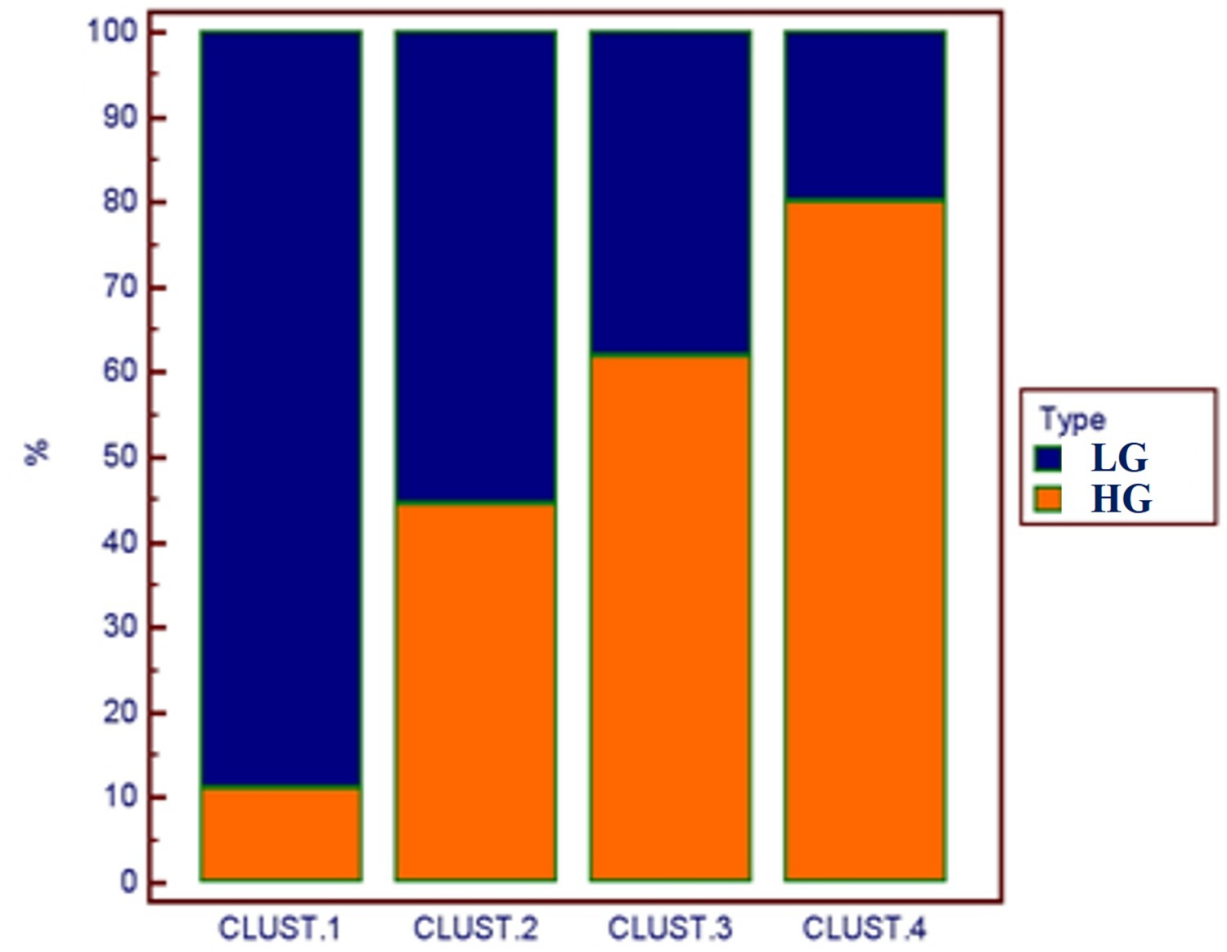
Histotype distribution in clusters. Unsupervised clustering analysis performed on gene expression values of CD44, E-cadherin, Survivin and CK20 in BCs revealed specific BC histotype distribution within clusters. LG: low grade; HG: high grade.

Considering the well-known heterogeneity of BC, we performed an Unsupervised Hierachical Clustering combining CD44, E-cadherin, Survivin and CK20 gene expression data. To perform the analysis it was necessary to have all four gene expression values for each sample so the total number of samples examined was reduced to 44 samples for the LG/HG analysis and to 30 samples for the grading and papillary morphology analysis. In Fig 7 is shown how gene expression profiles obtained by the combination of expression data revealed a similar trend between Cluster 2 and G2 group, and Cluster 4 and flat G3 group. The distribution of the two histotypes LG and HG in the four clustering groups was statistically significant with a p = 0.03 (Fig 8 and Table 7). On the other hand the distribution of grading (G1, G2 and G3), as well as the tumor morphology, in the four clusters was not statistically significant. Cluster 1 and Cluster 4 are the most distant groups and Cluster 1 contains a higher number of LG cases vs HG cases while Cluster 4, vice-versa, contained a higher number of HG cases.

**Table 7:** Distribution of BC samples within clusters (1–4).

	CLUSTER 1	CLUSTER 2	CLUSTER 3	CLUSTER 4		
**LG**	1	4	13	4	22 (50%)	
**HG**	8	5	8	1	22 (50%)	
**total**	9(20.5%)	9 (20.5%)	21 (47,7%)	5 (11,4%)	44	**p-value** 0.036
**G1**	1	3	1	1	6 (20%)	
**G2**	2	1	3	0	6 (20%)	**p-value** 0.1
**G3 papillary**	0	1	5	0	6 (20%)	
**G3 flat**	0	2	6	4	12 (40%)	
**total**	3 (10%)	7 (23%)	15 (50%)	5 (17%)	30	**p-value** 0.09

Distribution of BC samples on the basis of histotype, grading and papillary morphology in the four clustering groups created by the unsupervised hierarchical clustering analysis by using the combinations of gene expression values of CD44, E-cadherin, Survivin and CK20. LG: Low Grade; HG: High Grade.

## Discussion

BC is the 9^th^ most common cancer diagnosis worldwide [2] It is a heterogeneous disease, both clinically and pathologically, and its detection is based on methods with low sensitivity. Approximately 70% of all BCs are non-invasive low grade (LG) BC (Ta/pT1/CIS), typically growing as superficial papillary protrusions, genetically associated to FGFR3 mutations and characterized by a high risk of recurrence together to a rare (10–15%) ability to progress to high grade (HG) and invade. This capability seems to be likely due to TP53 acquired mutations. The remaining 30% of tumors are high grade (HG), flat and genetically associated to TP53 mutations. They develop from severe dysplasia or carcinoma in situ (CIS) and directly progress to become invasive (pT2-4). They are associated to resistance to cancer therapy and poor prognosis [5, 6, 7]. Predicting which LG tumor can progress and become invasive is a key goal for improving the patient outcome. TNM, grade and stage systems are not sufficient to accurately predict BC evolution. However, grade seems to be the better prognostic indicator of BC progression and mortality: 50% and at least 25% of CIS and pTaG3 respectively evolves becoming invasive [15]. Then main medical need, still unresolved, is to identify molecular markers that may translate into diagnostic or prognostic tools [16]. Here, we focused on the most promising prognostic markers.

First, we observed that HG and LG BCs did not show any significant association with TP53 and/or FGFR3 somatic mutations (Tables 5 and 6), without supporting the literature [17, 18] which reports TP53 and FGFR3 strictly related to HG and LG respectively. This discrepancy could be explained by the very small size of our study population suggesting the importance of validating these findings in a larger cohort of samples. Nevertheless, conflicting results have already been published concerning TP53 and FGFR3 mutation frequency in BC, as urderlined by Neuzillet Y et al (2012) that show how stage and grade can act as confusion factors creating spurious associations between the risk of each mutations. Only large study population, including all BC grades and stages, allows for properly adjusting association analysis between TP53 and FGFR3 [19]. Moreover, looking at the high frequency of TP53 mutation in LG group, we can’t exclude that our LG study population includes a copious number of the high propensity LG tumors able to evolve to HG ones (mainly associated to TP53 mutations). Indeed, within LG tumors, it is not actually possible to discriminate at diagnosis those who are able to acquire invasive features. On this perspective, mutations on TP53 gene could represent an early genetic event necessary to drive the 15% of LG BCs able to acquire invasive phenotype. On the other hand, in according with the literature, we found TP53 and FGFR3 as almost mutual exclusive mutations [17]: only 2/27 LG and 1/31 HG tumors showed co-occurrence of TP53-FGFR3 mutations. Regarding the morphology of HG tumors, our data show a higher frequency (about two folds) of TP53 mutations in flat tumors compared to papillary ones. This is in according with the literature, underling that HG tumors characterized with a high propensity to invade (such us flat HG) are mainly associated to TP53 mutations [5, 6]. In addiction, HG showed significant Survivin over-expression compared to LG non-invasive tumors, sustaining the role that Survivin could play, not only as a predictive biomarker for BC progression [20], but also as a potential therapeutic target. Moreover, we observed that Survivin expression seems to be inversely related to TP53 mutational status both in LG and HG BCs. In particular, within LG tumors, TP53 mutations (alone or in combination with FGFR3 mutations) were associated to a low Survivin mRNA levels compared to wild type BCs. In details, TP53 mutated BCs showed a 12.7 to 17.5 fold decrease of Survivin mRNA levels compared to wild type (data not significant) and FGFR3 mutated BCs (p = 0.021) respectively (Fig 3A). Regarding the grading, Survivin was significantly overexpressed in G3 compared with G1 (p = 0.04), maintaining the clear trend associated to the aggressive state of BC. Although not statistically significant, we found a 4 fold decrease of CK20 mRNA level in HG compared to LG BC, supporting the well-known heterogeneity of CK20 expression: in BC CK20 positive cells ranges from 15% to 97% [21]. Moreover, in the study conducted by Bassily et al, expression of CK20 was reported across all grades: 75% (3/4) were low malignant potential, 83% (5/6) LG, 38% (3/8) organ-confined HG, and 67% (6/9) high-grade tumors that invaded adjacent structures [22]. E-cadherin and CD44 data showed a trend already supported by the literature [23, 24]. CK20, CD44 and E-cadherin mRNA levels were not statistically associated to G1, G2 or G3 BC likely due to the small size of our study population.

However, among all the correlations identified (Fig 4), we underline the significant positive correlation between Survivin and CD44 in both, TP53 wild type (p = 0.0242) and FGFR3 wild type (p = 0.0036) tumors, and in FGFR3 wild type HG BCs (p = 0.0045). These results, not described before in the literature, suggest the role that Survivin and CD44 could play together in tumor progression, especially in those TP53-FGFR3 wild type BCs. Survivin and CD44 represent two important key factors for cancer development: Survivin is a crucial protein playing a role in apoptosis inhibition, whereas CD44 is a well known stemness marker associated to resistance to treatments [25]. Besides prognostic role, Survivin and CD44 could also represent focal targets for cancer therapy, blocking the progression especially in the TP53-FGFR3 wild type BCs able to become invasive [26].

Moreover, on the basis of the histological and the morphological diagnosis, we described and molecular characterized three BC sub-populations: LG with papillary morphology (G1+G2), HG papillary G3 and flat G3 (CIS). Indeed, Survivin showed a statistically significant up-regulation (7.7 fold) in flat G3 tumors compared to papillary LG group (p = 0.017) and papillary G3 tumors (p = 0.016) (Fig 6), while no difference was found between papillary LG and papillary G3. These results suggest that HG BC with higher propensity to progress, as the flat G3 (CIS) tumors, could be molecularly distinguished. According to the literature [27, 28], E-cadherin data describes an interesting trend negatively correlated to BC progression (Fig 6C), although not statistically significant, likely due to the low number of analyzed samples. CK20 and CD44 were also not statistically different among groups due probably, as well, by the small size of the analyzed population.

Considering the molecular heterogeneity we found in our BC population, we decided to exploit our gene expression data to perform an unsupervised hierarchical clustering data. We decided to use the unsupervised procedure to free the clustering software from any prior knowledge of the dataset, and to be able to explore the output data groups and to find retrospectively a biological meaning. The combination of the gene expression values of CK20, CD44, E-cadherin and Survivin genes was able to distinguish four clustering groups (Fig 7), which contained a progressive increasing number of HG cases going from Cluster1 to Cluster4 in which 80% of the cases were HG (Fig 8). Vice-versa 88% of Cluster1 was composed of LG cases. The distribution of the two histotypes was statistically significant with a p-value of 0.036 (Table 7). The use of an unsupervised clustering makes our data stronger, since no prior bias was included in the analysis and our results gives increasing importance to the use of a molecular classification for stratifying cancer populations. On the other hand no statistical significant distribution about histology and tumor morphology was found between the clusters, probably due by the very small size of our study population. It is important to underline that the grouping we obtain with few markers does not reflect the one that could be obtained by a whole-genome expression data analysis.

In conclusion, our study confirms FGFR3 and TP53 as mutual exclusive mutations, and underlines the significant negative correlation between Survivin gene expression and TP53 mutational status in both LG and HG tumors. We suggest here Survivin not only as a prognostic marker, in accordance to Jeon C et al [29] which showed the prognostic role of Survivin in BCs (by a meta-analysis approach), but overall as a potential therapeutic target to block, especially in TP53 wild type tumors. Indeed, interestingly, our study focuses exclusively on non-invasive BCs at diagnosis, showing Survivin statistically related to HG lesions and indicative of BC with high potential to invade (such as CIS). In addition, for the first time, we also report a significant positive correlation between Survivin and CD44 in TP53-FGFR3 wild type tumors, suggesting the potential role they could play, not only as predictors but also as therapeutic targets, especially by blocking the progression of the TP53-FGFR3 wild type BCs able to invade. These findings are to be further investigated by functional studies. Finally, we show by clustering analysis how is crucial to compare the histopathological approach to the molecular one, which is able to identify subgroups that otherwise could not be revealed. In the long run, the perspectives of studies like ours should aim at the translation of the molecular findings into clinically meaningful biomarkers. Stratifying the patients in more specific and characterized molecular groups will enable to establish the clinical relevance of each BC, give a better prognosis and develop personalized therapeutic strategies.

## Supporting information

S1 Text Supplementary materials and methods. DNA and RNA extraction, TP53 and FGFR3 gene mutational analysis and gene expression analysis of Survivin, CK20, CD44 and E-cadherin.
(DOCX)

S1 Tables Supplementary tables. Tumor features (Table A), Distribution of TP53 and FGFR3 mutations (Table B) and mRNA levels of Survivin, CK20, CD44 and E-cadherin (Table C).
(XLSX)
