# Supplementary material for: Molecular characterization of low grade and high grade bladder cancer
Source: PLoS One. 2019 Jan 16;14(1):e0210635. doi: 10.1371/journal.pone.0210635 (PMC6334926; doi:10.1371/journal.pone.0210635)
Supplement: S1 Text — DNA and RNA extraction, TP53 and FGFR3 gene mutational analysis and gene expression analysis of Survivin, CK20, CD44 and E-cadherin. (DOCX) [file pone.0210635.s001.docx]

**SUPPLEMENTARY MATERIALS AND METHODS**

**DNA and RNA extraction**

DNA and RNA were extracted from 4 x 10 µm FFPE tissue sections. Fresh tissue were stored in RNA-later solution (Ambion, Austin, TX) to avoid the RNA degradation until tissue homogenizion by GentleMACS dissociator (Miltenyi GmbH, Germany). DNA was isolated using Nucleospin Kit (Macherey-Nagel) and RNA extraction was performed by automatic system (Helix Extraction System, Diatech), according to the manufacturing instructions.

**TP53 and FGFR3 gene mutational analysis**

TP53 exon 4-9 PCR and FGFR3 exon 7, 10, 15 PCR were performed in 30 µl final volume, 1.25 Units of TaqGold (Applied Biosystem, MA) 0.1- 1µM of forward and reverse primers. Each amplification was performed for 35 cycles. PCR product was purified by Multiscreen HTS vacuum manifold system (Merck Millipore, Molsheim, France) and cycle sequencing using BigDye Terminator v3.1 (Applied Biosystem, MA) and analyzed by ABI-XL 3130 Sequencer (Applied Biosystem, MA). Because of the poor quality of the DNA extracted from the FFPE tissues we could not perform the TP53 and FGFR3 mutational status on all the samples.

**Gene expression analysis of Survivin, CK20, CD44 and E-cadherin**

Total RNA was reverse transcribed in cDNA in a final volume of 20 μl, containing 5X RT buffer, 10 mM dNTPs, 50 ng/μl Random Primers, 0.1M DTT, 40 U/μl RNaseOUT, 50 μM oligo(dT), DEPC-Treated Water, 15 U/μl Cloned AMV reverse transcriptase (Invitrogen, Carlsbad, CA). Gene expression levels were analysed by quantitative Real-Time PCR (qRT-PCR) on Rotor-Gene 6000 (Qiagen, Valencia, CA) following the manufacturing instructions. Endogenous reference gene (beta 2 microglobulin) was used to normalize each gene expression level. qRT-PCR was performed in 25 µl final volume, containing 5 µl of cDNA, 12.5 µl of MESA GREEN qPCR MasterMix Plus (EUROGENTEC, San Diego, CA), 300 nM of each primer (Invitrogen, Carlsbad, CA). Standard curves were generated for each gene to evaluate primer efficiency and for data analysis. To verify primers specificities, melting curve analysis was performed. For each experiment a no-template reaction was included as a negative control. Because of the poor quality of the RNA extracted from the FFPE tissues we could not perform the gene expression analysis on all the samples.
